# Image Restoration Using Functional and Anatomical Information Fusion with Application to SPECT-MRI Images

**DOI:** 10.1155/2009/843160

**Published:** 2009-10-01

**Authors:** S. Benameur, M. Mignotte, J. Meunier, J. -P. Soucy

**Affiliations:** ^1^Department of Computer Science and Operations Research (DIRO), University of Montreal, CP 6128l, Station Centre-Ville, P.O. Box 6128, Montréal, QC, Canada H3C 3J7; ^2^McConnell Brain Imaging Centre, Montreal Neurological Institute, 3801 University Street, Montréal, QC, Canada H3A 2B4

## Abstract

Image restoration is usually viewed as an ill-posed problem in image processing, since there is no unique solution associated with it. The quality of restored image closely depends on the constraints imposed of the characteristics of the solution. In this paper, we propose an original extension of the NAS-RIF restoration technique by using information fusion as prior information with application in SPECT medical imaging. That extension allows the restoration process to be constrained by efficiently incorporating, within the NAS-RIF method, a regularization term which stabilizes the inverse solution. Our restoration method is constrained by anatomical information extracted from a high resolution anatomical procedure such as magnetic resonance imaging (MRI). This structural anatomy-based regularization term uses the result of an unsupervised Markovian segmentation obtained after a preliminary registration step between the MRI and SPECT data volumes from each patient. This method was successfully tested on 30 pairs of brain MRI and SPECT acquisitions from different subjects and on Hoffman and Jaszczak SPECT phantoms. The experiments demonstrated that the method performs better, in terms of signal-to-noise ratio, than a classical supervised restoration approach using a Metz filter.

## 1. Introduction

In the image restoration framework, the regularization term is crucial to incorporate knowledge concerning *a priori* acceptable solutions and to constrain the solution of this ill-posed inverse problem. In this paper, we are concerned with image restoration issues using information from a different modality (intermodality) for this fusion-based regularization term. More precisely, we herein propose an original extension of the NAS-RIF restoration technique allowing the NAS-RIF inverse filtering to be constrained from an intermodality registration, that is, a registration between anatomical and the functional medical images to be restored (from the anatomical structure of the same patient). In our application, anatomical information is extracted from a high-resolution anatomical procedure such as magnetic resonance imaging (MRI) or computed tomography (CT) and this high spatial resolution modality is exploited to improve the contrast of functional SPECT images. 

 The contrast and signal-to-noise ratio displayed by brain single photon emission computed tomography (SPECT) images is rather limited, when compared with that from anatomical techniques (MRI, CT scanning). This fact limits the potential use of brain SPECT images. For instance, it is not easy to differentiate low tracer uptake due to a functional deficit, where brain tissue still is anatomically intact, from low uptake generated by focal atrophy, where tissue is lost and replaced by cerebrospinal fluid (CSF) [1]. 

 Up to now, several methods have been proposed to improve directly or indirectly the spatial resolution of SPECT images. These methods can be split into two major classes: those using restoration techniques during the reconstruction process from projections, and those where the restoration is performed on the already reconstructed images. In this paper, we are describing a posttomographic reconstruction process, an approach which has the advantage of being essentially independent from the physical features of the scanner. 

 In this category, we can cite [2] where Rajabi et al*.* compared four widely used filters (i.e., Hanning, Butterworth, Metz and Wiener) for myocardial ^99*m*^Tc-sestamibi SPECT perfusion studies. In [3] a nonnegativity and support constraints recursive inverse filtering (NAS-RIF) algorithm proposed by Kundur and Hatzinakos [4], was extended to the 3D SPECT imaging restoration context. The NAS-RIF blind deconvolution technique is relevant to any situations in which a finite object of interest is imaged against a uniformly grey (or noisy) background [4]. This method can thus be efficiently exploited in brain SPECT imaging since the true undistorted rCBF map of a human brain consists of a finite support imaged against a noisy background (the background depending of both the Poisson noise phenomenon inherent to imaging with radioactive elements and to other, nonPoisson sources of background in the images such as electronic noise from the scanner). The only information required for this deconvolution procedure is the nonnegativity of the true image and the support of the object to be restored. In [3], this support was accurately determined by an unsupervised 3D Markovian segmentation technique applied to the SPECT volume. 

 Using the same strategy, but this time during the reconstruction process, we can cite Bayesian tomographic reconstruction methods [5, 6] which use structural information on the presence and location of important anatomical “landmarks” (such as local discontinuities or extended homogeneous regions as seen for instance on an MR anatomical image) to control noise without smoothing edges. These models usually express that, within a detected and segmented “uniform” anatomical region, neighboring pixels in the functional image should have similar grey level values (local homogeneity) or follow a Gaussian distribution with a unique mean value (global homogeneity) [7, 8]. 

 In this paper, we propose to extend the method presented in [3] by introducing into the NAS-RIF algorithm a new spatially-adaptive regularization term for SPECT image deconvolution. This regularization term allows to efficiently include anatomical information extracted from a high-resolution anatomical MRI image [9] while stabilizing the solution of the NAS-RIF inverse filter by preventing noise amplification and ringing artifacts. In our application, this structural anatomy-based regularization term exploits the result of an unsupervised Markovian segmentation obtained after a preliminary registration step between the MRI and SPECT volumes from the same patient. The proposed regularization term is quadratic and the NAS-RIF procedure thus involves recursive filtering of the degraded image to minimize a newly convex objective cost function with a conjugate gradient method. Our restoration method was tested on 30 pairs of brain MRI and SPECT images from different patients and on Hoffman and Jaszczak SPECT Phantoms and compared with a standard supervised deconvolution/restoration approach using a classical Metz filter. 

 This paper is organized as follows. Section 2 briefly describes the proposed 3D anatomical constraint version of the NAS-RIF deconvolution technique. Section 3 describes the registration and segmentation algorithms. Section 4 presents the validation protocol of the new restoration method. We then show some of our experimental results on phantom and real brain SPECT volumes and validate the proposed model in Section 5. Finally, we conclude in Section 7.

## 2. 3D Anatomical Constraint NAS-RIF Algorithm

### 2.1. 3D Extended Version of the NAS-RIF

In our application, and as proposed in [3], we assume that 3D SPECT images are degraded according to the following, widely-used linear model:

(1) g(x,y,z)=f(x,y,z)*h(x,y,z)+n(x,y,z),

in which *g*(*x*, *y*, *z*), *f*(*x*, *y*, *z*), and *h*(*x*, *y*, *z*), respectively, denote the degraded 3D image, the true image and the point spread function (PSF). *n*(*x*, *y*, *z*) represents the additive noise and ∗ designates the 3D discrete linear convolution operator. The 3D blind deconvolution problem consists then in determining *f*(*x*, *y*, *z*) and *h*(*x*, *y*, *z*) (or its inverse) given the blurred observation *g*(*x*, *y*, *z*). 

 In the 3D extended version of the NAS-RIF deconvolution strategy, the output of the FIR filter *u*(*x*, *y*, *z*) of dimension *N*_*xu*_ × *N*_*yu*_ × *N*_*zu*_ gives an estimate of the true image $\hat{f}(x,y,z)$. Each resulting estimation is passed through a nonlinear filter which uses a nonexpansive mapping to project the estimated 3D image into the space representing the known characteristics of the true image (expressing in fact that the image is assumed to be nonnegative with a known support). The difference between this projected image ${\hat{f}}_{\text{NL}}$ and $\hat{f}$, *e*(*x*, *y*, *z*), is used as the error signal to update the variable filter *u*(*x*, *y*, *z*). In the 3D context, the cost function used in the deconvolution procedure of the 3D image is defined as

(2) $$\begin{array}{c} J\left(u\right)=J_{1}\left(u\right)+J_{2}\left(u\right)+\gamma \;J_{3}\left(u\right) \end{array}$$

with

(3) $$\begin{array}{c} J_{1}\left(u\right)=\underset{\left(x,y,z\right)\in 𝒟}{\sum }{\hat{f}}^{2}\left(x,y,z\right)\left(\frac{1-\mathrm{sgn}\;\left(\hat{f}\left(x,y,z\right)\right)}{2}\right),\\ J_{2}\left(u\right)=\underset{\left(x,y,z\right)\in \bar{𝒟}}{\sum }{\left(\hat{f}\left(x,y,z\right)-L_{\text{B}}\right)}^{2},\\ J_{3}\left(u\right)={\left(\underset{\forall \left(x,y,z\right)}{\sum }u\left(x,y,z\right)-1\right)}^{2}, \end{array}$$

where $\hat{f}(x,y,z)=g(x,y,z)*u(x,y,z)$, and sgn(*f*) = −1 if *f* < 0 and sgn(*f*) = 1 if *f* ≥ 0. *𝒟* is the set of all pixels of *g*(*x*, *y*, *z*) inside the region of support, and $\bar{𝒟}$ is the set of all pixels outside the region of support. 

 The first term, *J*_1_(*u*), is used to penalize negative voxels in the support in order to keep the image estimate nonnegative. The second term *J*_2_(*u*) penalizes voxels located outside the support with values which deviate significantly from the background average *L*_B_. When the background of the true image is black, that is, *L*_B_ = 0, the third term, *J*_3_(*u*), is used to avoid a trivial all-zero minimum solution (*γ* being a positive constant). 

The authors have shown in [10] that the above equation is convex in the 2D case with respect to *u*. This property remains true in the 3D case so that convergence of the algorithm to the global minimum is ensured using the conjugate gradient minimization routine [10].

### 2.2. Anatomical Constraint 3D NAS-RIF

The major shortcoming of the NAS-RIF technique is its noise amplification at low SNR [4]. This is due to the high-pass property of the inverse filter *u*(*x*, *y*, *z*) which amplifies high-frequency noise. As a result, the solution at convergence may not be the best estimate of the original object in the presence of noise. In order to solve this problem, a solution has been suggested by Kundur and Hatzinakos [4], which consists in halting the iterative restoration process through visual inspection. In practice, this requires a strong supervision and, even in this case, it is not so easy to determine which is the optimal iteration for termination (different parts of the image may converge at different rates), making this method unreliable. 

 In this work, we propose an alternative regularization approach for the NAS-RIF algorithm which can also be viewed as a way to incorporate anatomical information extracted from a (high-resolution) anatomical MRI (or CT) image into the SPECT data. The proposed regularization term also allows stabilization of the inverse solution by preventing noise amplification, does not require supervision (parameters tuning or stopping criterion) and is capable of introducing better constraints on the solution of our restoration problem. This strategy consists in applying, over predetected and segmented anatomical regions, a piecewise smoothness constraint on the functional SPECT image to be recovered. To this end, our regularization term exploits the result of a preliminary registration step between the MRI and SPECT images as well as the result of a segmentation of the MRI image into anatomical classes. 

 In our model, the new cost function related to the deconvolution of the 3D image is now defined as

(4) $$\begin{array}{c} J\left(u\right)=J_{1}\left(u\right)+J_{2}\left(u\right)+\gamma \;J_{3}\left(u\right)+\delta \;J_{4}\left(u\right) \end{array}$$

with:

(5) $$J_{4}\left(u\right)=\overset{3}{\underset{i=1}{\sum }}\;\underset{\left(x,y,z\right)\in r_{i}}{\sum }{\left(\hat{f}\left(x,y,z\right)-{\bar{r}}_{i}\right)}^{2},$$

where the first summation is made on the three main “anatomical” types (tissues) found in the brain, that is, white matter (*r*_**WM**_), grey matter (*r*_**GM**_), and cerebro-spinal fluid (*r*_**CSF**_) and ${\bar{r}}_{i}$ designates the mean, in grey levels, of the *i*th region and *δ* is a weighting factor between this anatomical constraint and the hard constraints of the NAS-RIF procedure. In this context, *𝒟* = *r*_**WM**_ ∪ *r*_**GM**_ ∪ *r*_**CSF**_ and ${\bar{r}}_{i}=(1/N_{r_{i}})\sum_{(x,y,z)\in r_{i}}\hat{f}(x,y,z)$ where *N*_*r*_*i*__ is the number of voxels in the region *r*_*i*_. 

*J*_4_(*u*) is proportional to the sum of variance within each anatomical region (for each transversal slice) of the SPECT image. This term expresses that, within a detected and segmented anatomical region, pixels in the functional image in general tend to have similar grey level values. This regularization term is edge-preserving since it allows to apply a smoothness constraint, while preserving (anatomical) discontinuities. 

 Furthermore, the introduction of this regularization term *J*_4_ does not affect the convexity of the NAS-RIF cost function, and therefore a unique solution to the problem is still guaranteed. Figure 2 shows the structure of this scheme. A coregistered SPECT and (high-resolution) MRI datasets “along with the result of a segmentation of the MRI volume into anatomical classes” are used as input for the cost function. 

 The first derivative of the cost function in (4) is shown in (7). The gradient vector of *J* with respect to *u* is:

(6) $$\nabla J\left(u\right)=(\frac{\partial J\left(u\right)}{\partial u\left(0,0,0\right)}\;\cdots \;\frac{\partial J\left(u\right)}{\partial u\left(i,j,l\right)}{\;\cdots \;\frac{\partial J\left(u\right)}{\partial u\left(N_{xu}-1,N_{yu}-1,N_{zu}-1\right)})}^{T},$$

where each entry is expressed as:

(7) $$\begin{array}{ll} & \frac{\partial J\left(u\right)}{\partial u\left(i,j,l\right)}\\ & \;=2\underset{(x,y,z)\in 𝒟}{\sum }\hat{f}\left(x,y,z\right)\left(\frac{1-\mathrm{sgn}\;\left(\hat{f}\left(x,y,z\right)\right)}{2}\right)\\ & \;\;\times g\left(x-i,y-j,z-l\right)\\ & \;\;+2\underset{(x,y,z)\in \bar{𝒟}}{\sum }\left(\hat{f}\left(x,y,z\right)-L_{\text{B}}\right)g\left(x-i,y-j,z-l\right)\\ & \;\;+2\gamma \;\left(\underset{\forall (x,y,z)}{\sum }u\left(x,y,z\right)-1\right)\\ & \;\;+2\delta \overset{3}{\underset{n=1}{\sum }}‍\;\underset{(x,y,z)\in r_{n}}{\sum }\left(\hat{f}\left(x,y,z\right)-\frac{1}{N_{r_{n}}}\underset{(x,y,z)\in r_{n}}{\sum }\hat{f}\left(x,y,z\right)\right)\\ & \;\;\times \left(g\left(x-i,y-j,z-l\right)-\frac{1}{N_{r_{n}}}\underset{(x,y,z)\in r_{n}}{\sum }g\left(x-i,y-j,z-l\right)\right). \end{array}$$

A gradient-based iterative restoration algorithm or its conjugate version can be efficiently applied to minimize this convex cost function. Besides, since the proposed criterion is quadratic, many other optimization methods can be used. 

 The initial inverse FIR filter required by the NAS-RIF algorithm is the Kronecker delta function [10]; the size of this inverse filter is set to 3 × 3 × 3 pixels. Furthermore, we have used *γ* = 0 because the background of SPECT images is not completely “black” [4]. 

 Finally, the convergence criterion of the proposed algorithm is the stability of the cost function to be minimized, that is,

(8) $$\begin{array}{c} \frac{J\left(u^{\left[l+1\right]}\right)-J\left(u^{\left[l\right]}\right)}{J\left(u^{\left[l\right]}\right)}\leq \epsilon , \end{array}$$

where *ϵ* is a threshold, typically set in our application to 10^−3^, with the upper-script denoting the iteration number. Figure 3 shows the evolution of the cost function value along the iterations of the gradient descent process for the image restoration presented in Figure 8. 

 In order to define our anatomically based regularization term *J*_4_, we exploit the result of a 3D registration step between the MRI and SPECT input volumes (from the same patient) [11, 12] and then an unsupervised Markovian segmentation of the (registered) MRI 3D image into anatomical classes [9] (See Section 3.2).

## 3. Registration and Segmentation

In order to define our anatomically based regularization term *J*_4_, we exploit the result of a 3D registration step between the MRI and SPECT input volumes (from the same patient) and then an unsupervised Markovian segmentation of the (registered) MRI 3D image into anatomical classes.

### 3.1. Registration

The 3D registration method used in our application is based on mutual information (MI) and is fully described in [12]. The MI registration criterion *C*(*θ*) between the input MRI and SPECT volumes describes the amount of information in the joint histogram of the images; hence its maximization results in the best match of intensity correspondences between the images for registration. The optimal set of registration parameters *θ*_optimal_ is then found by maximizing *C*(*θ*), where the vector *θ* is simply estimated by the Powell's method [13]. The images are smoothed slightly in order to make the cost function *C*(*θ*) as smooth as possible to give faster convergence and less chance of finding bad local minima (related to a wrong registration). The code used to register the MR image to the SPECT image is mainly inspired from the software package Statistical Parametric Mapping (SPM) [14]. (See Figure 7).

### 3.2. Segmentation of the MRI Volume

To this end, we consider two random fields (*X*, *G*), where *G* = {*G*_*s*_, *s* ∈ *S*} represents the field of observations located on the 3D lattice *S* of sites *s* (voxels) and *X* = {*X*_*s*_, *s* ∈ *S*} the label field (related to the class labels *X*_*s*_ of a segmented 3D image). Each aforementioned label is associated to a specific brain “tissue” category or region on the 3D image. CSF and extra-cerebral tissues are combined in a single class, corresponding to tissue without (significant) tracer uptake. Although skin and other structures outside the brain actually have a nonzero tracer presence (they have blood flow), we assume this to be negligible here. The CSF area designates regions within the brain and immediately around it that are actually devoid of activity (intracerebal ventricles and peri-cerebral cysterns). The white matter and grey matter (brightest region) are associated with lower and higher levels of blood flow, respectively, [15]. Each *G*_*s*_ takes its value in {0,…, 255} (256 grey levels), and each *X*_*s*_ in {*e*_1_ = “CSF”, *e*_2_ = “whitematter”, *e*_3_ = “greymatter”}. The distribution of (*X*, *G*) is defined, firstly, by a prior distribution *P*_*X*_(*x*), assumed to be Markovian and secondly, by the site-wise conditional data likelihoods *P*_*G*_*s*_/*X*_*s*__(*g*_*s*_/*x*_*s*_) whose shape and parameter vector Φ_(*x*_*s*_)_ depend on the concerned class label *x*_*s*_ (*g*_*s*_ designates the grey level intensity associated to the site *s*).

**Estimation Step**

In order to determine Φ = (Φ_(*e*_1_)_, Φ_(*e*_2_)_, Φ_(*e*_3_)_), we use the Iterative Conditional Estimation (ICE) algorithm [16] and three different Gaussian laws for the likelihood.

**Segmentation Step**

Based on the estimates given by the ICE procedure, we can compute an unsupervised 3D Markovian segmentation of the MR volume. In this framework, the Markovian segmentation can be viewed as a statistical labeling problem according to a global Bayesian formulation in which the posterior energy has to be maximized [17]:

(9) $$U\left(x,g\right)=\underset{U_{1}(x,g)}{\underset{︸}{\underset{s\in S}{\sum }-\ln\;P_{G_{s}\;|\;X_{s}}(g_{s}\;|\;x_{s})}}+\underset{U_{2}(x)}{\underset{︸}{\underset{\langle s,t\rangle }{\sum }\beta_{st}\;\;(1-\delta (x_{s},x_{t}))}}$$

where *U*_1_ expresses the adequacy between observations and labels, and *U*_2_ represents the energy of the *a priori* Pott model (which tends to favor homogeneous regions with no privileged orientation). *β*_*st*_ (= 1 here) is a weight parameters. We use the deterministic Iterated Conditional Modes (ICM) algorithm [17] to minimize this global energy function. For initialization of this algorithm, we use the segmentation map obtained by a Maximum Likelihood (ML) segmentation. The support *𝒟* is then determined simply by the set of pixels belonging to CSF, white and grey matter classes. (See Figures 5  and 6).

## 4. Validation

### 4.1. SPECT Data Acquisition and Reconstruction

The SPECT images were acquired with a triple-head gamma camera (Picker Prism, Cleveland, OH, USA) equipped with low-energy, high-resolution parallel-holes collimators. The SPECT projections were acquired over 360°. 90 projections of 50 seconds each were obtained. The radioisotope was ^99*m*^Tc-ECD (Ethylene Cysteinate Dimer).

### 4.2. MRI Data Acquisition

MRI images were acquired on a Siemens Magnetom Avanto 1.5T scanner using a 3D-FISP with a radial trajectory in *k*-space. It used a nonselective excitation. The scanning parameters were TR = 9.2 ms, TE = 2.2 ms with *N* slices of 512 × 512 voxels with voxel dimensions of 0.5 × 0.5 × 1.0 mm^3^, and *N* ∈ [130, 150]. These 3D MRI images were further processed to isolate the brain from other tissues, using the brain extraction tool (BET) [18] of the MRIcro software by adjusting BET's fractional intensity threshold.

### 4.3. Validation Protocol on Phantoms

Two imaging phantoms were considered in order to validate the accuracy of our SPECT images restoration method

**Figure 1:**
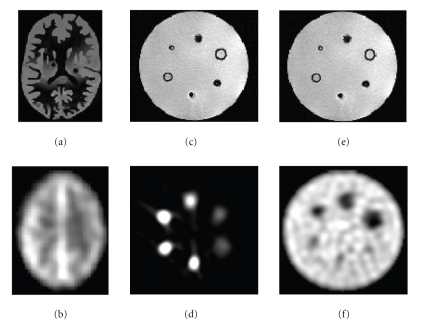
Transaxial slices of the phantoms. (a) Hoffman phantom MRI. (b) Hoffman phantom SPECT. (c) Cylindrical phantom MRI. (d) Cylindrical phantom SPECT with hot spheres. (e) Cylindrical phantom MRI. (f) Cylindrical phantom SPECT with cold spheres.

**Figure 2:**
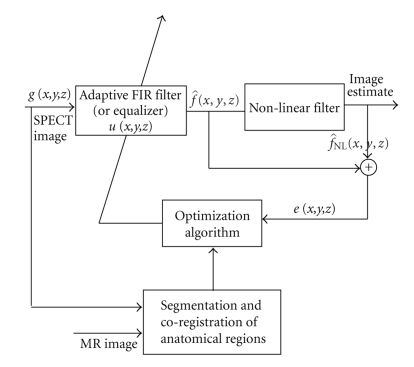
Three-dimensional extension of the NAS-RIF deconvolution algorithm with incorporation of anatomical constraints.

**Figure 3:**
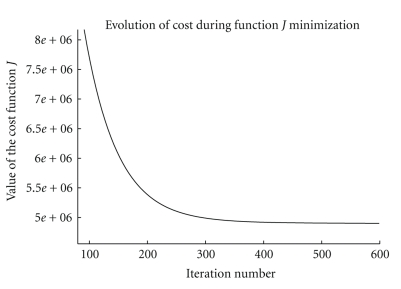
Evolution of the cost function *J* along the iterations of the gradient descent process for an image restoration.

- For SPECT imaging, the Hoffman 3D Brain Phantom [19] was scanned while containing 148 MBq of activity. The phantom was positioned so that the orientation of the slices within the phantom would match as much as possible those from the MRI image. Phantom SPECT data included a set of 61 slices of 128 × 128 voxels with voxel dimension of 1.85 × 1.85 × 1.85 mm^3^. The MRI Phantom data contained 209 slices of 256 × 256 voxels with 1 mm isotropic voxels. Figure 1 shows transversal slices of the Hoffman phantom.
- The other phantom contains 6 spheres of different sizes (diameters #1: 9.5 mm, #2: 12.7 mm, #3: 15.9 mm, #4: 19.1 mm, #5: 25.4 mm, #6: 31.0 mm). In one condition, the spheres were filled with a radioactive solution and the rest of the cylinder (background) with a less concentrated solution of the same radioisotope, giving an activity concentration ratio (spheres/background) of approximately 2.7 : 1; in another condition, the cylinder was filled with a low-activity solution and the spheres were filled with nonradioactive water. Phantom SPECT data included a set of 93 slices of 128 × 128 voxels with voxel dimensions of 1.85 × 1.85 × 1.85 mm^3^. The Phantom MRI data contained 224 slices of 256 × 256 voxels with 1 mm isotropic voxels. Figure 1 shows transaxial slices of the phantom (Deluxe ECT) [20].

Simple visual examination is an easy method for evaluation of the restorative power of a technique, but it is obviously an insufficient approach. A better evaluation approach consists in computing a performance measure based on the improvement in signal-to-noise ratio (ISNR), expressed in decibels (dB), using both the degraded phantom, the ground truth (or the original undegraded image given by the MRI image), and the restored phantom images. The ISNR is defined by [21],

(10) $$\text{ISNR}=10\;{\log\;}_{10}\left(\frac{{\left\|I_{\text{ori}}-I_{\deg\;}\right\|}_{2}}{{\left\|I_{\text{ori}}-I_{\text{res}}\right\|}_{2}}\right),$$

where *I*_*deg*_ is a given degraded phantom image, *I*_ori_ is the corresponding original (ground truth) phantom image and *I*_res_ is the restored phantom image. || · ||_2_ is a symbol for quadratic norm. Obviously, this metric can only be used with knowledge of the original object; in our case this came from the MRI phantom and knowledge of the radioactivity concentration within each subcompartment of the phantom. 

 In addition, restored images were also evaluated by the specific evaluation criteria proposed in [22, 23], based on the estimation of the four following measures:

- the global contrast [22] of the image, defined by *C*_*G*_ = (1 − *m*_**WM**_/*m*_**GM**_), where *m*_**WM**_ and *m*_**GM**_ are the means of the pixel value in the white and grey matter areas respectively.
- the local contrast of the image [23], defined by *C*_*L*_ = (*R*_*i*_ − *B*_*j*_)/*B*_*j*_, where *R*_*i*_ represents the mean grey level value inside the *i*th sphere and *B*_*j*_ represents the mean grey level value outside the *i*th sphere (in a circle centered around the sphere and whose radius *D* is half the distance from one sphere center to the next in the image.)
- the image mottle *M*_**WM**_ in the white matter region [22], defined by *M*_**WM**_ = *σ*_**WM**_/*m*_**WM**_, where *σ*_**WM**_ is the standard deviation of pixel values in this area.
- the image mottle *M*_**GM**_ in the grey matter region [22], defined by *M*_**GM**_ = *σ*_**GM**_/*m*_**GM**_, where *σ*_**GM**_ is the standard deviation of pixel values in this area.

These two metrics *M*_**WM**_ and *M*_**GM**_ allow us to measure the amplification of noise and/or to measure the presence of undesirable artifacts that could be created by the restoration procedure in a uniform region of the SPECT volume. Due to the different number of pixels belonging to each brain anatomical tissue category, we considered the total mottle measure given by *M* = *ρ*_**WM**_ *M*_**WM**_ + *ρ*_**GM**_ *M*_**GM**_, with *ρ*_**WM**_ and *ρ*_**GM**_ designating the proportion of pixel belonging to the white and gray matter area respectively. A reliable SPECT image restoration method is one that enhances image contrast with little increase in the mottle. Inversely, for a given maximal mottle measure, we can measure if the contrast enhancement is significant [22].

### 4.4. Comparison with a Supervised Metz Restoration Filter

We have compared our blind and unsupervised deconvolution approach to a classical deconvolution technique using a Metz filter [24]. The Metz filter is a supervised deconvolution (restoration) procedure which assumes knowledge of the point spread function (PSF) of the imaging system. The filter is a combination of an inverse filter and a low pass filter. This filter allows deconvolution of SPECT images while attenuating very high frequencies (i.e., noise which could be induced by inverse filtering) [24]. Please remember that the Metz filter has two parameters to be adjusted, the full width at half maximum (FWHM) related to the filter size and *p* (which is the order of the filter).

## 5. Experimental Results

Restoration of clinical data was performed on pairs of MRI and SPECT images from 30 epileptic patients. Each SPECT data set contained *N* slices of 128 × 128 voxels with voxel dimensions of 1.85 × 1.85 × 1.85 mm^3^, and *N* ∈ [69, 103]. MRI data sets contained *M* slices of 512 × 512 voxels with voxel dimensions of 0.5 × 0.5 × 1.0 mm^3^ and *M* ∈ [130, 150]. In this section, a few examples from that group are presented. 

 Isolation of the brain (in MRI) from other tissues was made *off-line* prior to the registration and restoration steps. BET's fractional intensity threshold was fixed at 0.50. This value was chosen empirically after a series of test runs which were visually evaluated. 

**Figure 4:**
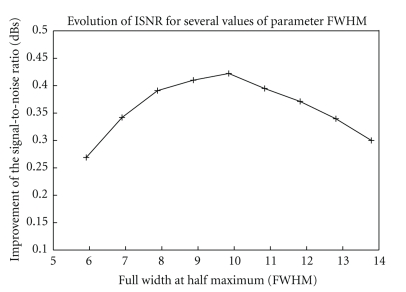
ISNR as a function of the parameter FWHM for the Hoffman phantom SPECT.

**Figure 5:**
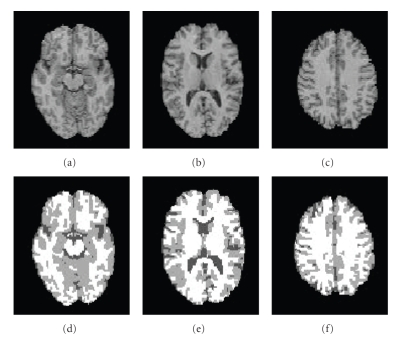
Examples of human brain MRI cross-sectional images. (a), (b), (c): Original MRI cross-sectional human brain images. (d), (e), (f): Unsupervised three-dimensional Markovian segmentations.

**Figure 6:**
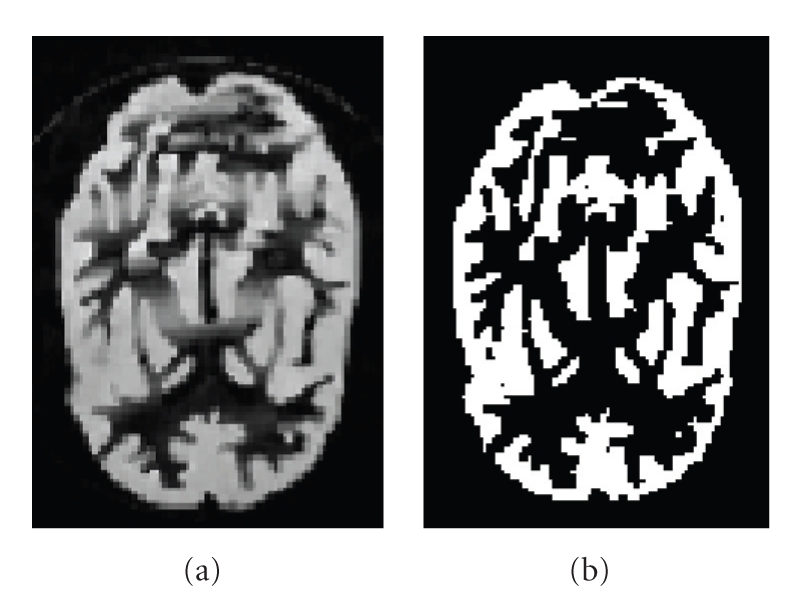
Example of segmentation for phantom. (a) Original MRI. (b) Segmented MRI.

**Figure 7:**
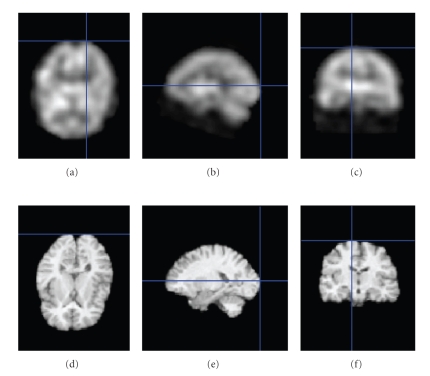
Examples of registration of the MR volume to the SPECT volume (using registration method described in Section 3.1). (a), (b), (c): Axial, sagittal, and coronal view of a SPECT volume. (d), (e), (f): Axial, sagittal, and coronal view of a registered MR volume.

Figure 4 shows the variation in ISNR of the processed phantom images with respect to the observations for a range of FWHM parameter and for the best value of *p*. By examining the ISNR for different values of FWHM, we noticed that the optimal restoration value was for FWHM = 9.85 mm, *p* = 40 corresponding to ISNR = 0.42 dB. 

 Average contrast and total mottle were first quantified on a set of (human brain) degraded and restored SPECT images (with and without our anatomical-based regularization term *J*_4_(*u*)) and compared to the Metz filter. Our proposed algorithm with *J*_4_(*u*) resulted in an increase of global contrast by 1.87 and of mottle by an acceptable factor of 1.17. The Metz filter increased global contrast by 1.52 and mottle by a factor of 1.08. 

 For SPECT phantoms, the results are shown in Table 1 and give similar results. For all the tested experiments, we used *γ* = 0 because the background of SPECT images is not completely “black” [4] and we chose 25 for the weighting factor *δ* for *J*_4_. This value was chosen empirically after a set of experiments by varying *δ* in the interval [10, 100].

 SPECT images of the cold spheres phantoms were also restored using partially incorrect anatomic information (i.e., cylindrical phantom MRI with 5 different sphere sizes instead of 6; one sphere was removed in the MRI segmented image). Table 2 shows that the local contrast measured on this restored SPECT image (with our anatomical-based regularization term *J*_4_(*u*)) remained similar to the local contrast obtained with correct anatomic information. 

 Local contrast was quantified on individual spheres of SPECT degraded and restored images (with and without our anatomical-based regularization term *J*_4_(*u*)) and compared to those obtained with the Metz filter. Table 3 shows that our algorithm with *J*_4_(*u*) resulted in a average value of the increase in local contrast of 2.07. The Metz filter increased, in average, the local contrast by only 1.53. 

**Figure 8:**
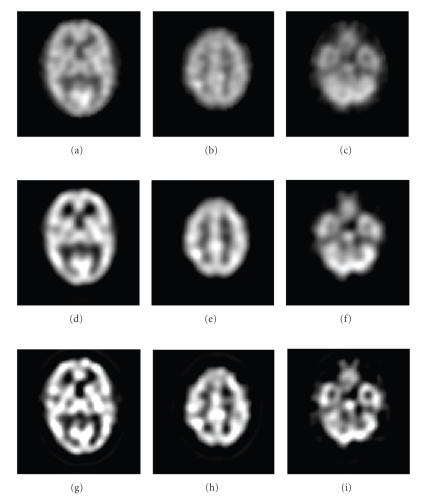
Examples of deconvolutions for the Hoffman phantom. (a), (b) and (c): Original SPECT cross-sectional images. (d), (e) and (f): deconvolution result with Metz filter. (g), (h) and (i): deconvolution result with our method.

**Figure 9:**
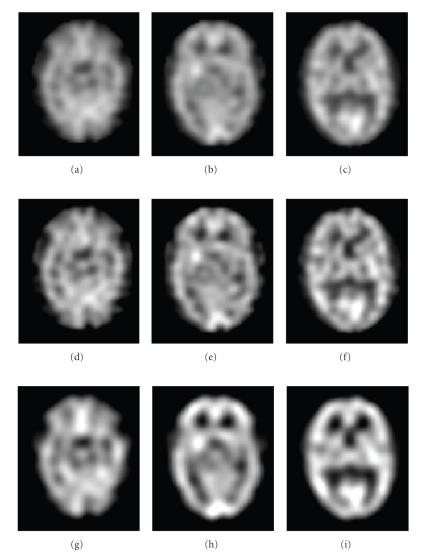
Restoration results. Axial view of: (a), (b) and (c): Original SPECT cross-sectional images. (d), (e) and (f): deconvolution result with Metz filter. (g), (h) and (i): deconvolution result with our method.

 Figures 8  and 9  show, respectively, examples of the Hoffman phantom and brain SPECT volumes obtained with our restoration method. The reconstructed whole brain volume converged to a very good estimate of the solution without * a priori* information about the PSF and allowed to noticeably improve the contrast of the original, unrestored SPECT volume. This restoration should allow more efficient detection of small, localized singularities associated with different types of lesions (tumors, epileptogenic foci, etc.) that often are not clearly visible in the original blurred image. 

 The Metz filter has two parameters to be adjusted, *FWHM* and order *p*. We have used the values of 4.5, 5, 5.5, 6, 6.5, 7, 8, 9, 10 and 11 mm for *FWHM* and 10, 20, 30, 40 and 50 for order *p*, a total of 50 combinations of both parameters. All the above values were tested and only the best one (in terms of *ISNR*) was selected for comparison with our algorithm. 

 This study was IRB (institutional review board) approved. In addition, the 30 pairs of brain MRI and SPECT acquisitions from different subjects have been anonymised using Jim's DICOM anonymiser tool. (This tool allows the removal from image files of information that may jeopardise patient's or physician's privacy.)

## 6. Discussion

Our restoration procedure was not highly sensitive to the BET's fractional intensity threshold value when this was visually set by an experimented user (e.g., the value of contrast was stationary when the threshold went from 0.45 to 0.55). Therefore, this parameter was set to 0.50 for all thirty pairs of MRI images and seemed to be optimal in all tested cases. 

 Notice that the degradation model used by our restoration method assumes that noise is additive while in reality it is a multiplicative Poisson process. Nevertheless, the results with real SPECT/MRI data are visually impressive. It seems that at the high count level used in SPECT, the difference between (multiplicative) Poisson and (additive) Gaussian noise does not affect significantly the efficiency of the algorithm. However it would be meaningful to conduct clinical studies using ROC analysis to better assess this restoration procedure. These clinical studies will be the topic of an another medical article. 

 When compared with a classical restoration approach using a Metz filter, our method performed better, in terms of signal-to-noise ratio. The slight increase in mottle with *J*_4_ is a limited price to pay given the gains in global contrast, ISNR, and local contrast measures, which from a clinical perspective ensure better detection of focal anomalies, a task at which SPECT is usually notoriously poor. 

 The tests also showed that the restored image is constrained (or guided) by our prior knowledge but not wrongly biased by this information while avoiding noise amplification inherent to each deconvolution process. 

 The computational time of our technique was approximatively 6.33 minutes as compared to 0.24 minutes for the Metz filter on a 2.0 GHz PC workstation running Linux. Our method is computationally demanding but remember that it is an unsupervised one which does not require any PSF parameter. 

 Another disadvantage of our method is the need to get an MRI scan; however, many of the subjects submitted to a SPECT rCBF study actually require one from a clinical perspective. Importantly, the accuracy of the registration procedure is crucial for the final restoration result, fortunately many highly accurate registration procedures are widely available elsewhere.

## 7. Conclusion

In this paper, we have presented a robust restoration method using anatomical and functional information fusion with application to SPECT images. This method improves the quality of human brain 3D SPECT images and should thus be helpful to physicians interpreting such studies. Our approach takes advantage of the anatomical information contained in the MRI study of each subject (or any other high-resolution image such as CT). The proposed constraint term allowed both stabilization of the inverse solution of the NAS-RIF procedure by prevention of noise amplification and the generation of a better constraint on the solution of our restoration problem. In the regularization framework, this term allowed smooth regions to be reconstructed in the SPECT image, where such homogeneous anatomical regions are found in the high-resolution MRI images of the patient, after those were registered to the subject's SPECT volume. This method was tested on a number of SPECT/MRI pairs, demonstrating its efficiency and robustness. This 3D blind restoration technique is completely data driven and automated, therefore it could be implemented to process large numbers of 3D SPECT studies.

## Figures and Tables

**Algorithm 1:**
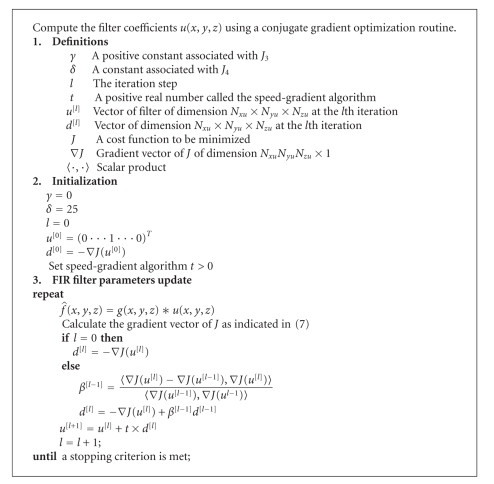
NAS-RIF Algorithm.

**Table 1:** The global contrast *C*_*G*_ and the mottle *M* (expressed in %) and the improvement signal-to-noise ratio **ISNR** (expressed in dB) obtained from, respectively, the restored image with and without *J*_4_(*u*), the Metz filter, and the degraded images that are the original phantom SPECT.

Phantoms	Restored images									Degraded images	
	with *J*_4_(*u*)			Metz filter			without *J*_4_(*u*)				

	*C*_*G*_	*M*	*ISNR*	*C*_*G*_	*M*	*ISNR*	*C*_*G*_	*M*	*ISNR*	*C*_*G*_	*M*

Hoffman	30.4	25.2	0.720	23.0	20.1	0.420	19.2	17.8	0.298	18.2	17, 4
Cold spheres	28.2	36.4	0.680	20.7	32.3	0.398	19.5	26.5	0.240	17.0	26.0
Hot spheres	27.8	38.7	0.669	20.1	33.1	0.385	19.0	27.4	0.226	16.3	26.4

**Table 2:** Local contrast *C*_*L*_ (expressed in %) with and without correct anatomic information obtained from the restored image with *J*_4_(*u*).

Cold Sphere_*i*_	1	2	3	4	5	6
C_*L*_ (with partially incorrect anatomic information)	22.7	20.0	17.3	14.6	10.6	5.6
C_*L*_ (with correct anatomic information)	23.3	19.5	15.9	14.8	9.4	6.9

**Table 3:** The local contrast *C*_*L*_ (expressed in %) with correct anatomic information obtained from, respectively, the restored image with and without *J*_4_(*u*), the Metz filter, and the degraded image that are the original phantom SPECT.

	Restored images			Degraded
Cold sphere_*i*_	with *J*_4_(*u*)	Metz filter	without *J*_4_(*u*)	image

	*C*_*L*_	*C*_*L*_	*C*_*L*_	*C*_*L*_

1	23.3	17.2	16.1	14.4
2	19.5	16.7	14.9	12.1
3	15.9	12.3	11.2	9.3
4	14.8	10.4	9.0	7.8
5	9.4	7.1	6.0	4.1
6	6.9	4.6	3.1	2.1
